# Lost to Follow-Up After Patent Foramen Ovale (PFO) Closure: Late Detection of Abdominal Aortic Embolization of an Occluder Device

**DOI:** 10.7759/cureus.102632

**Published:** 2026-01-30

**Authors:** Amir Hakanovic, Mirza Kovacevic, Minela Hadzic Abdurahmanovic, Igal Moarof

**Affiliations:** 1 Department of Cardiology, Mittelland Centre for Cardiology, Aarau, CHE; 2 Department of Anesthesia and Critical Care, Zenica Cantonal Hospital, Zenica, BIH; 3 Department of Medicine, University of Zenica, Zenica, BIH; 4 Department of Internal Medicine, Regional Hospital Spital Zofingen AG, Oftringen, CHE

**Keywords:** abdominal aortic embolization, amplatzer occluder, device embolization, long-term follow-up, patent foramen ovale closure

## Abstract

Embolization of a patent foramen ovale (PFO) occluder is a rare complication, particularly when detected years after device implantation. We report a case of a 30-year-old patient who initially presented with acute right lower limb ischemia caused by embolic occlusion of multiple arteries. A floating thrombus was detected in the inferior vena cava, and subsequent evaluation revealed a PFO, a bicuspid aortic valve, and an aortic arch anomaly. Six weeks later, PFO closure was performed using a 25 mm Amplatzer Cribriform device (Abbott, Chicago, Illinois), followed by appropriate post-procedural antithrombotic therapy. Nearly five years after implantation, routine follow-up revealed asymptomatic embolization of the occluder to the suprarenal abdominal aorta at the level of the superior mesenteric artery, without evidence of limb or renal hypoperfusion; the exact timing of embolization could not be determined. Percutaneous retrieval was attempted but aborted due to pain and the high risk of aortic injury, and the device was therefore left in situ. This case highlights a rare late complication of PFO closure and underscores the importance of long-term surveillance in patients with complex cardiovascular anatomy and thrombotic risk factors.

## Introduction

Patent foramen ovale (PFO) is a common congenital cardiac anomaly, present in approximately 15-35% of the adult population [1]. Although it is frequently asymptomatic and often detected incidentally, its clinical relevance lies in its potential role as a conduit for paradoxical embolism, enabling venous thrombi to enter the systemic circulation under conditions of transient or sustained right-to-left shunting [2]. This mechanism has been implicated in cryptogenic stroke, peripheral arterial embolism, and other systemic thromboembolic events, particularly in younger patients without traditional cardiovascular risk factors [3].

Percutaneous closure of PFO has therefore become an established preventive strategy in selected patients, supported by randomized trials demonstrating a reduction in recurrent embolic events compared with medical therapy alone [4]. The procedure is generally considered safe, with high technical success and a low rate of major complications. Reported adverse events are most commonly minor and occur in the peri-procedural or early post-procedural period, reflecting the overall favorable safety profile of contemporary occluder devices [5]. Among recognized complications, device embolization is rare (0.7-1.2%) and typically occurs shortly after implantation, most often related to suboptimal device sizing, inadequate anchoring within the interatrial septum, or complex septal anatomy [6, 7]. Consequently, early post-procedural imaging and short-term follow-up are emphasized in routine clinical practice. In contrast, late device embolization occurring months or years after an initially successful implantation is exceptionally uncommon and sparsely reported. Previously described sites of late embolization include the pulmonary artery, left atrium, left ventricle, ascending and descending aorta, and, less frequently, the abdominal aorta. Clinical outcomes vary widely, ranging from incidental, asymptomatic detection to acute limb ischemia, visceral hypoperfusion, or the need for urgent surgical or endovascular retrieval, underscoring both the rarity and potential clinical significance of this complication [8]. Current guidelines and consensus documents recommend structured follow-up after PFO closure, typically including transthoracic or transesophageal echocardiography within the first months after implantation to confirm device position and endothelialization, followed by periodic clinical surveillance thereafter [9]. However, beyond the first year, long-term follow-up intervals are less clearly defined and are often individualized, which may contribute to under-recognition of late complications. Moreover, unforeseen circumstances such as healthcare service disruptions during the COVID-19 pandemic have further challenged standard surveillance strategies. In many centers, routine follow-up imaging and outpatient evaluations - normally performed at predefined intervals during the first year and intermittently thereafter - were delayed or omitted, increasing the likelihood that late device-related complications remained undetected.

We present a case of a young adult who was lost to follow-up due to service disruptions related to the COVID-19 pandemic and experienced an asymptomatic embolization of a PFO occluder to the abdominal aorta, of unknown date. Our case illustrates key diagnostic considerations, management challenges, and long-term follow-up implications associated with the late detection of a PFO occluder embolized to the abdominal aorta.

## Case presentation

In 2020, a 30-year-old male patient with no prior comorbidities presented to the emergency department with intermittent claudication and a cool, pale right foot. Further investigations, including CT angiography, revealed subacute ischemia with embolic occlusion of the right profunda femoris artery, occlusion of the tibiofibular trunk and the distal anterior tibial artery, as well as occlusion of the proximal posterior tibial artery on the right side. In addition, a floating thrombus was detected in the inferior vena cava extending into the right common iliac vein, together with a paracentral pulmonary embolism on the right and a lower-lobe pulmonary embolism on the left.

The patient was admitted to the vascular surgery department, where angiography-guided embolectomy was performed using the Fogarty maneuver. On the first postoperative day, a re-embolectomy was required due to recurrent occlusion of the profunda femoris artery. Subsequent duplex examination confirmed patency of the profunda femoris artery.

**Figure 1:**
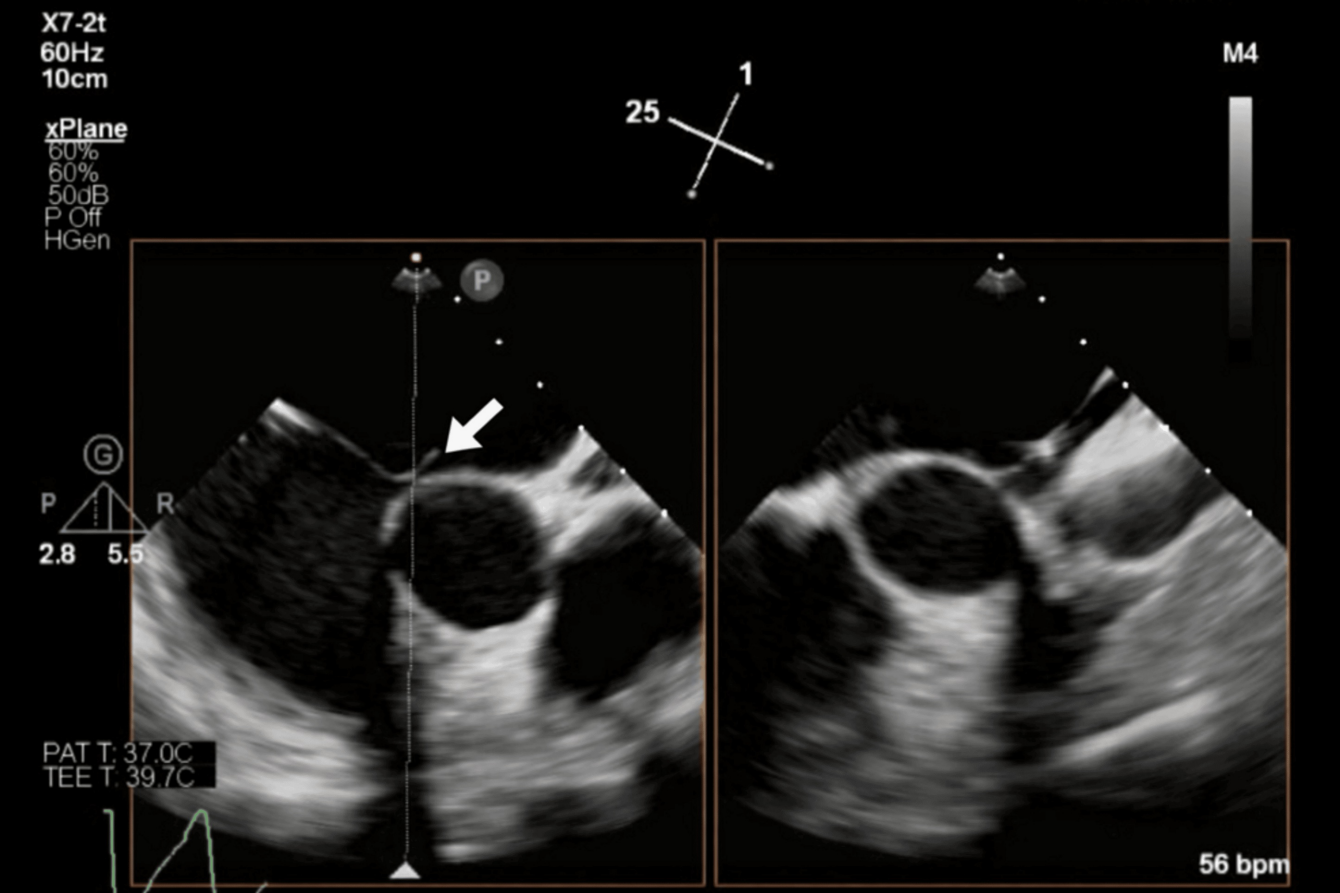
Transesophageal echocardiography performed during diagnostic workup Arrow indicated a large patent foramen ovale

**Figure 2:**
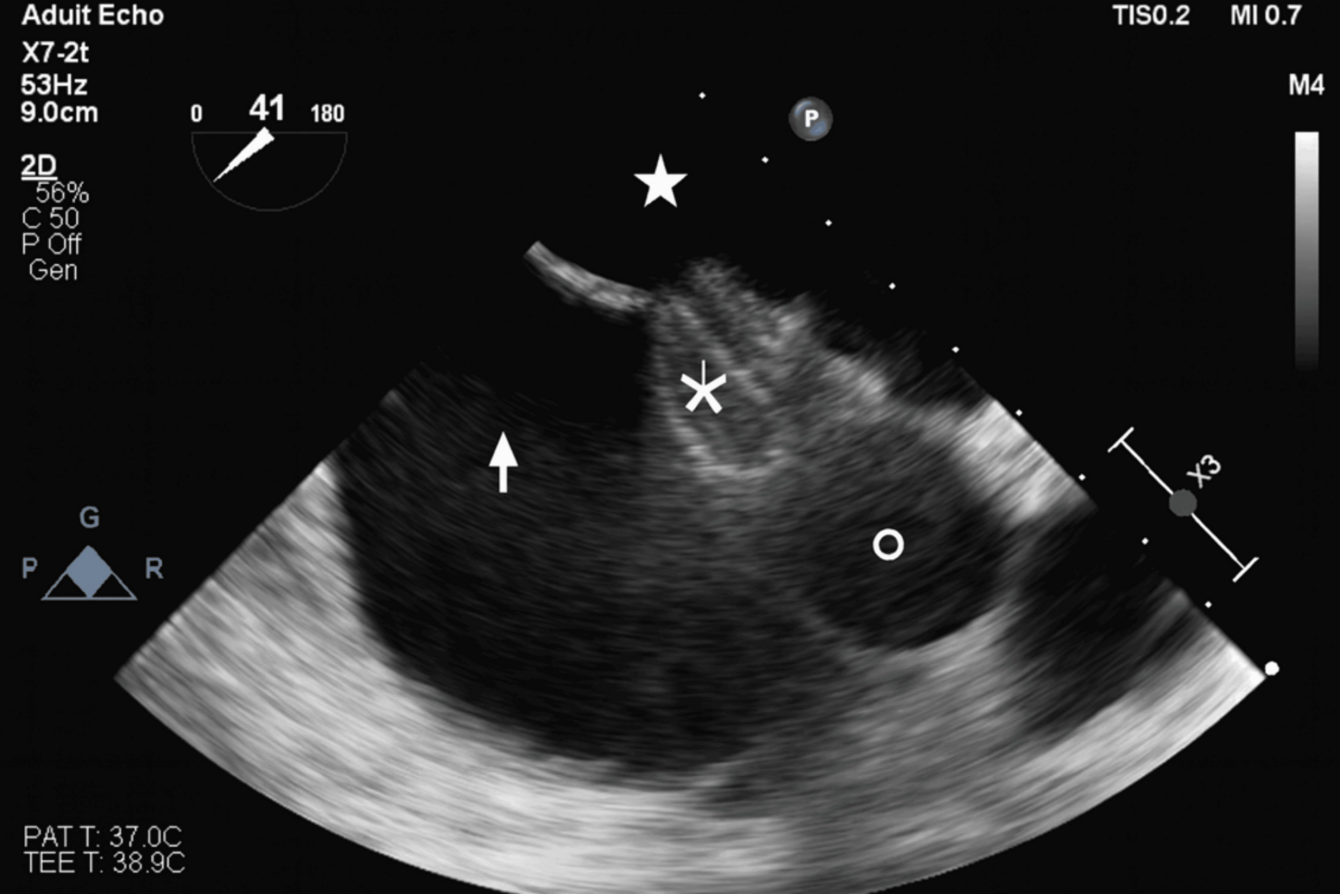
Intra-procedural TOE demonstrating correct PFO occluder deployment Arrow indicated right atrium, solid star indicates left atrium, snowflake indicates PFO occluder, and circle indicates aortic root. TOE - transesophageal echocardiography; PFO - patent foramen ovale

As no new embolic events occurred under therapeutic anticoagulation with low-molecular-weight heparin (dalteparin sodium 200 IU/kg body weight, totaling 15,000 IU over two weeks, followed by rivaroxaban 20 mg/day), placement of an inferior vena cava filter was deemed unnecessary. Cardiological workup with transthoracic and transesophageal echocardiography (TEE) revealed a PFO with an atrial septal aneurysm (protrusion of the atrial septum of 17 millimeters), which likely enabled paradoxical embolization of the thrombus into the left-sided circulation (Figure 1). Additionally, a bicuspid aortic valve (Sievers type 0) and an aortic arch anomaly with an aberrant right subclavian artery (arteria lusoria), as well as the left-sided carotid and vertebral arteries arising directly from the aortic arch, were identified. Hematological evaluation ruled out antiphospholipid antibody syndrome. However, a persistently elevated factor VIII level of 229%, exceeding the normal reference range (approximately 50-150%), was observed and likely contributed to an underlying thrombophilic state. Given the patient's history of a flu-like infection prior to the ischemic event, SARS-CoV-2 infection was ruled out. Atrial fibrillation was excluded in the outpatient setting. Six weeks later, percutaneous PFO closure was performed using a 25 mm Amplatzer Cribriform device (Abbott, Chicago, Illinois). A cribriform device was selected due to its larger left atrial disc compared with a standard PFO occluder, facilitating adequate coverage and closure of the PFO. Procedural echocardiographic monitoring confirmed correct device positioning (Figure 2). The patient was placed on dual antiplatelet therapy (DAPT) consisting of acetylsalicylic acid and clopidogrel for six months, followed by acetylsalicylic acid monotherapy. The planned follow-up, including routine transesophageal echocardiography at six months after PFO closure, was not performed due to service disruptions related to the COVID-19 pandemic.

**Figure 3:**
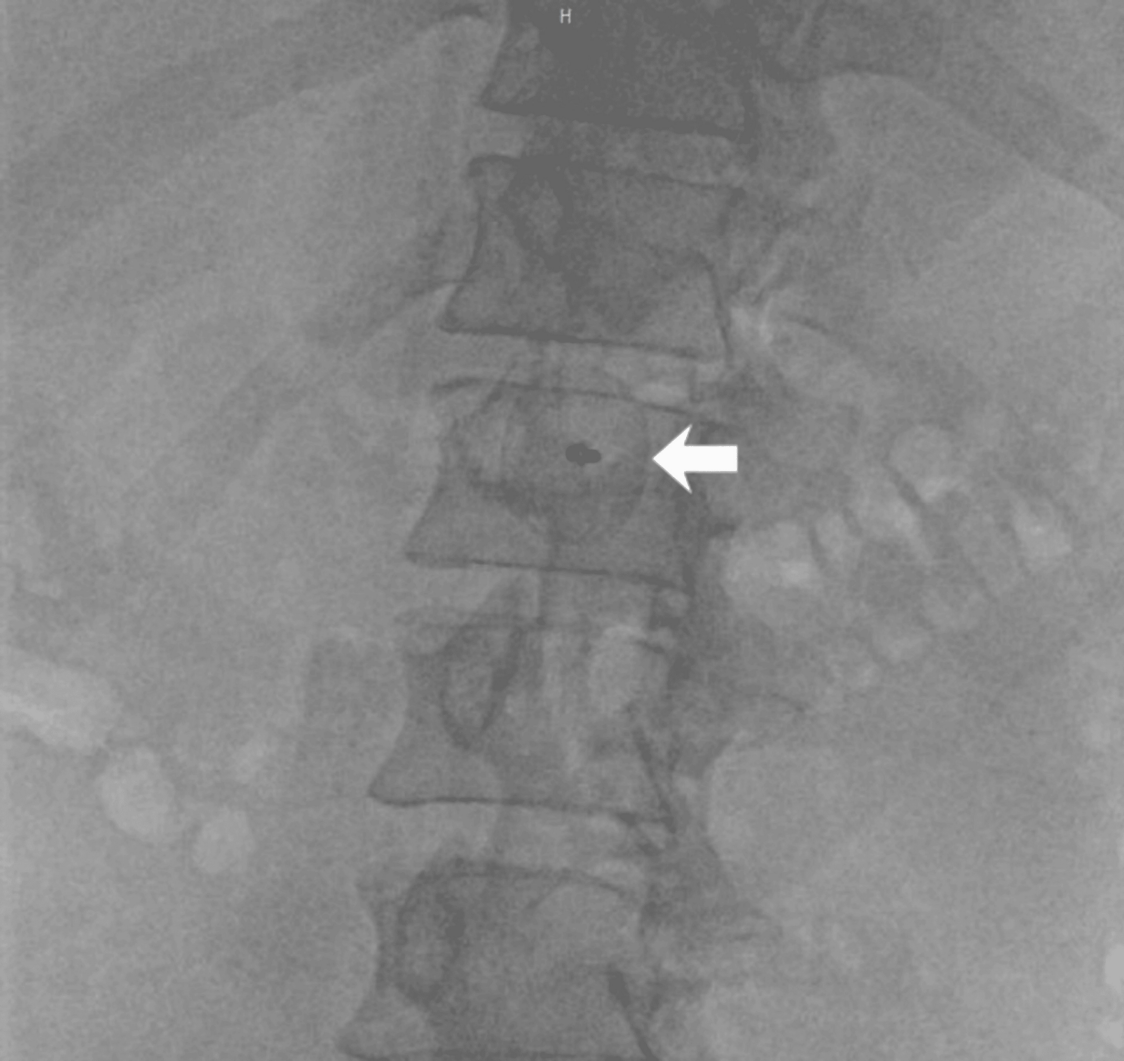
Fluoroscopy in the cardiac catheterization laboratory showing a PFO occluder in the abdominal aorta Arrow indicates PFO occluder PFO - patent foramen ovale

**Figure 4:**
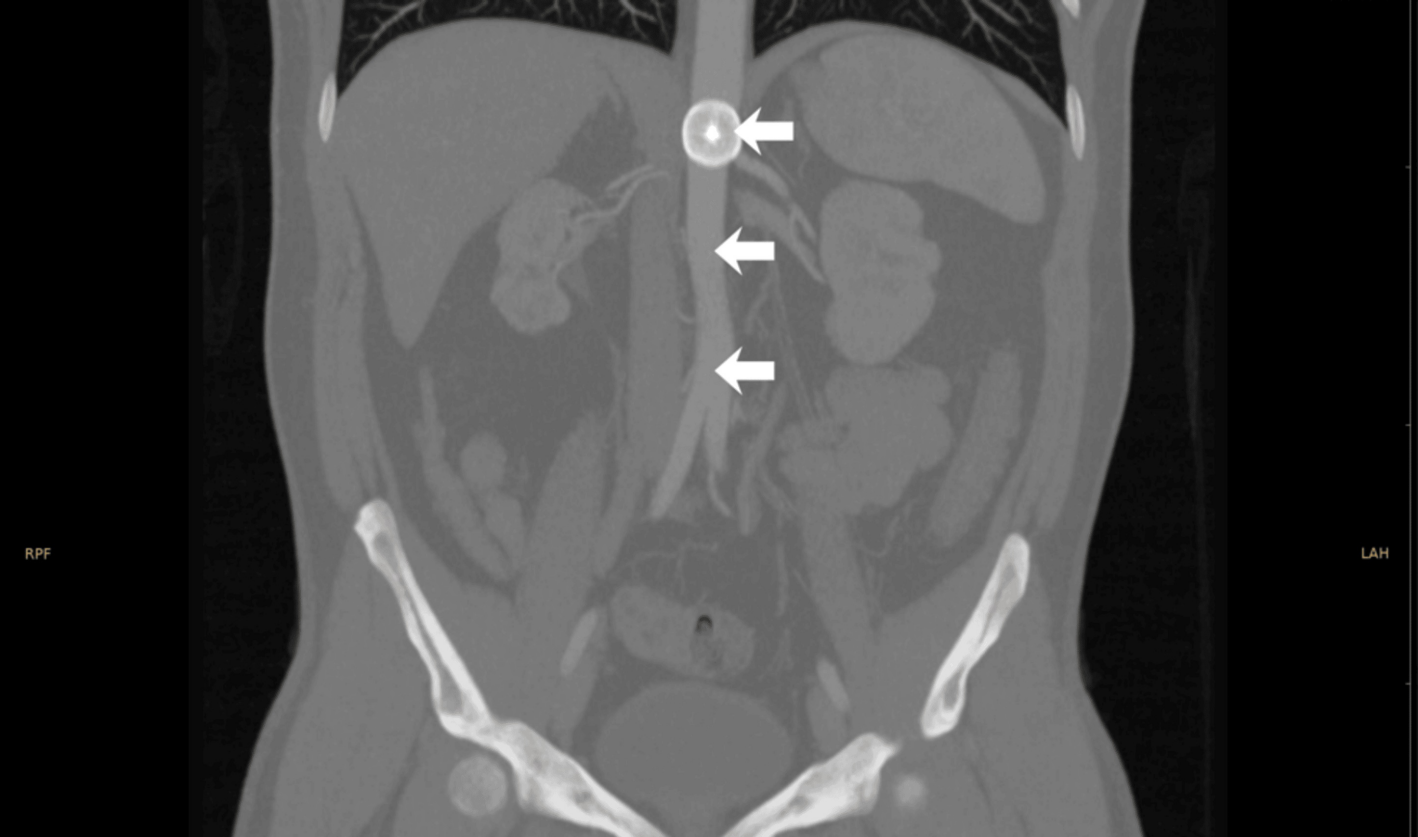
CT angiography demonstrating device embolization into the abdominal aorta Arrows indicate device embolus

**Figure 5:**
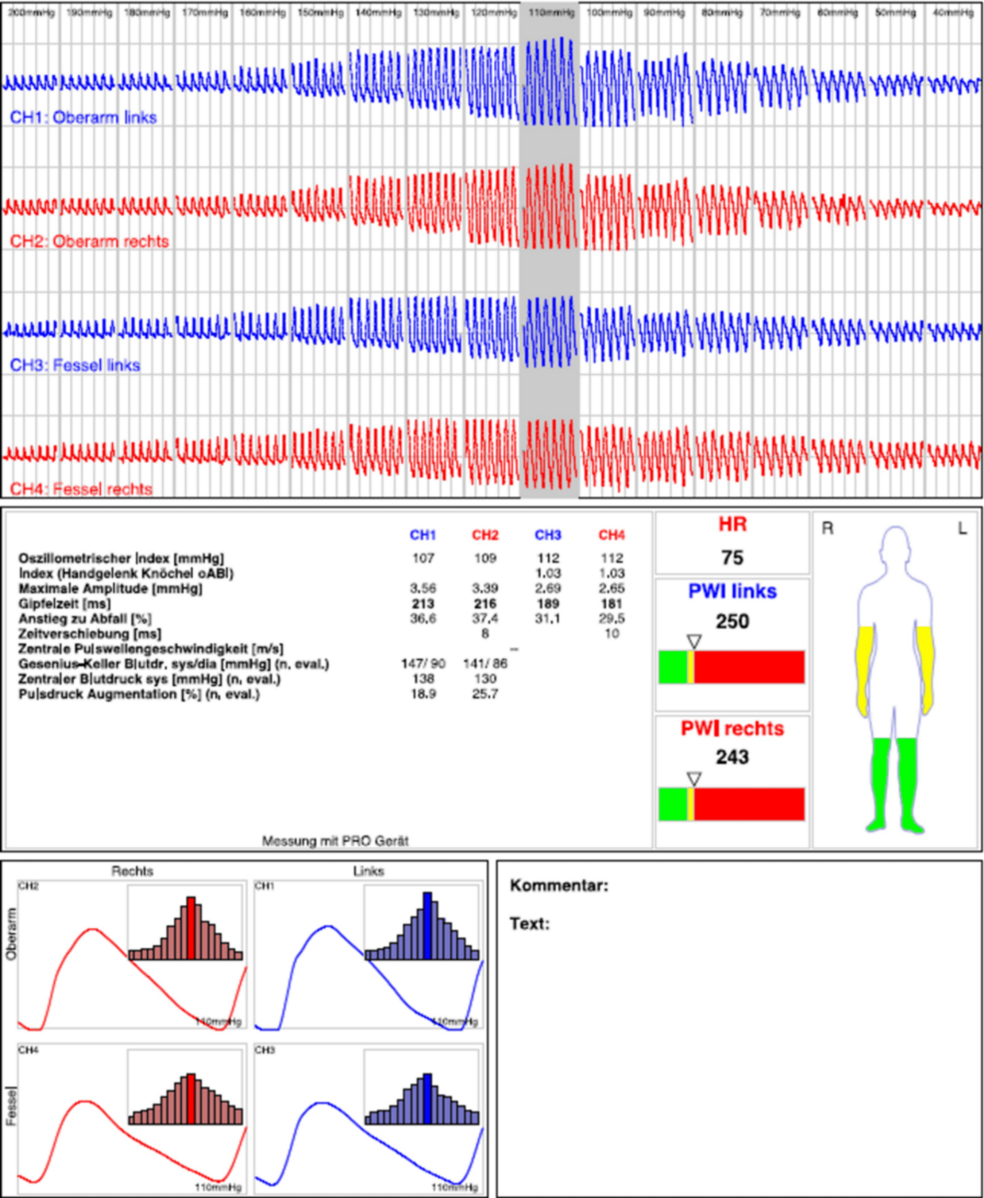
Oscillometric pulse-wave recordings of all four extremities demonstrating normal pulse-wave morphology, symmetric amplitudes, and indices within physiological ranges

Almost five years later, the patient returned for his first cardiology follow-up. Due to limited transthoracic acoustic quality, the PFO occluder could not be visualized. Fluoroscopy in the cardiac catheterization laboratory demonstrated that the occluder had embolized into the abdominal aorta (Figure 3). An abdominal CT angiogram confirmed embolization of the device into the abdominal aorta, immediately cranial to the renal artery origins, with the epicenter at the level of the superior mesenteric artery (Figure 4). Duplex sonography and oscillography confirmed normal macroperfusion of the lower extremities (Figure 5).

The renal artery branches were not clearly visualized on duplex sonography; however, there were no indirect signs of renal hypoperfusion (normal kidney size and normal renal function parameters). After interdisciplinary consultation, percutaneous removal of the occluder was recommended. The device was grasped using a snare catheter (Video 1). Even with gentle pulling, the patient experienced abdominal pain; therefore, further extraction was avoided due to the risk of aortic rupture in the absence of hemodynamically significant obstruction. As the occluder appeared endothelialized and there was no evidence of stenosis affecting the renal arteries or the superior mesenteric artery, it was decided to leave the device in situ. Regular duplex follow-up examinations are planned. Surgical removal will be considered if signs of hemodynamic relevance develop in the future.

**Video 1:** Embolized patent foramen ovale occluder captured with a snare catheter, followed by an attempted withdrawal

## Discussion

PFO closure is a widely used intervention to prevent paradoxical embolism, particularly in patients with cryptogenic stroke or systemic thromboembolism [10]. Device embolization is a rare complication; it typically occurs early after implantation [11]. Possible reasons for closure device migration include choosing an inappropriate device size, suboptimal positioning during deployment, tearing of the atrial septum caused by device manipulation, or limited operator experience. Additional reported contributors to device dislodgement and later migration are the presence of an atrial septal aneurysm, excessive septal mobility (excursion ≥10 mm), increased septum secundum thickness (>10 mm), a prominent Eustachian valve, or an elongated tunnel-like anatomy [12]. In our case, the most likely cause appears to be the atrial septal aneurysm, given that other echocardiographic features were not particularly remarkable. Embolization may involve partial or complete displacement of the device to the atria, the main pulmonary artery, or other locations within the vascular system. Once detached from its delivery cable, retrieval becomes challenging and can be life-threatening depending on the device's location [13]. Imaging with transesophageal echocardiography (TOE) is highly valuable for pre-procedural assessment of anatomical suitability and for detecting intracardiac thrombi [14].

In our patient, the embolized PFO occluder was identified nearly five years after the procedure due to a lapse in follow-up. The exact timing of the embolization remains unclear, although early embolization is suspected. The absence of symptoms allowed the embolized device to remain undetected for several years, highlighting the diagnostic challenges associated with such rare events [15]. To the best of our knowledge, it is remarkable that no adverse events, including paradoxical embolism, occurred over the five-year period despite massive thromboembolization at the index event. The embolized occluder was located in the suprarenal abdominal aorta, immediately cranial to the renal arteries, with its epicenter at the level of the superior mesenteric artery. Management of late PFO occluder embolization requires careful balancing of retrieval risks against potential end-organ compromise [16, 17]. Percutaneous removal was attempted but aborted due to patient discomfort and the risk of aortic injury. Given that the device was endothelialized and did not cause hemodynamically significant obstruction, conservative management with rivaroxaban 10 mg once daily and regular imaging follow-up was chosen after multidisciplinary team discussion. This approach aligns with available literature and published case reports suggesting that asymptomatic, non-obstructive embolized devices can be safely monitored, particularly when the procedural risks of retrieval are high [18].

This case emphasizes the importance of both short- and long-term follow-up after PFO closure, especially in patients with complex anatomy. Awareness of possible complications is crucial for timely recognition and individualized management, potentially preventing severe outcomes. Furthermore, the use of advanced imaging modalities, including CT or MRI angiography, may provide more precise information regarding device position, vessel wall interaction, and thromboembolic burden, and could guide future management decisions. Reporting such rare late complications contributes to the growing understanding of device behavior over extended periods and can inform best practices for monitoring and intervention.

## Conclusions

Late-detected embolization of a PFO occluder is a rare but clinically significant complication, particularly when asymptomatic. This case highlights both the general clinical implications of device embolization and the specific lessons learned from delayed detection. Awareness of this potential outcome, especially in patients with complex cardiovascular anatomy, is essential for timely recognition and appropriate management. Careful patient selection and appropriate device choice represent modifiable factors that may reduce the risk of late complications.

Careful long-term follow-up with structured imaging surveillance (Doppler ultrasound, oscillometric pulse-wave recordings, and laboratory assessment) can help identify embolized devices before they cause hemodynamic compromise or organ injury. Periodic echocardiographic assessment, with additional cross-sectional imaging when indicated, is recommended to ensure timely detection. Individualized management strategies, including conservative monitoring or intervention (anticoagulant therapy), should be guided by device position, patient symptoms, and procedural risks.

This case demonstrates that consistent follow-up is essential after PFO closure, as loss to follow-up can result in delayed recognition of major complications such as late device embolization and may directly influence patient safety, management decisions, and clinical surveillance strategies.
